# Worse Comes to Worst: Bananas and Panama Disease—When Plant and Pathogen Clones Meet

**DOI:** 10.1371/journal.ppat.1005197

**Published:** 2015-11-19

**Authors:** Nadia Ordonez, Michael F. Seidl, Cees Waalwijk, André Drenth, Andrzej Kilian, Bart P. H. J. Thomma, Randy C. Ploetz, Gert H. J. Kema

**Affiliations:** 1 Wageningen University and Research Center, Wageningen, The Netherlands; 2 Centre for Plant Science, The University of Queensland, Brisbane, Australia; 3 Diversity Arrays Technology, University of Canberra, Bruce, Canberra, Australia; 4 University of Florida, IFAS, Department of Plant Pathology, Tropical Research & Education Center, Homestead, Florida, United States of America; McGill University, CANADA

## Bananas: Their Origin and Global Rollout

The banana is the most popular fruit in the world and ranks among the top ten food commodities for Southeast Asia, Africa, and Latin America [1]. Notably, the crop is largely produced by small-holder farmers, with around 85% of the global production destined for local markets and only 15% entering international trade [1]. Bananas evolved in the Indo-Malayan archipelago thousands of years ago. The majority of all edible varieties developed from specific (inter- and intra-) hybridizations of two seeded diploid *Musa* species (*M*. *acuminata* and *M*. *balbisiana*) and subsequent selection of diploid and triploid seedless clones [2,3]. Despite rich genetic and phenotypic diversity [4], only a few clones developed, over time, into global commodities—either as dessert bananas, such as the triploid “Cavendish” clones, or as important staple foods such as cooking bananas and plantains [4,5]. Currently, bananas are widely grown in the (sub)tropics and are consumed in nearly all countries around the world, providing crucial nutrition for millions of people. Edible bananas reproduce asexually through rhizomes, but since the early 1970s, tissue culture has enabled mass production of cultivars [6]. This facilitates the rapid rollout of genetically identical plants, which have consumer-preferred traits and outstanding agronomical performance, onto vast acreages around the world. However, the typical vulnerability of monocultures to diseases has taken its toll on banana production over the last century. In 1876, a wilting disease of banana was reported in Australia [7], and in 1890, it was observed in the “Gros Michel” plantation crops of Costa Rica and Panama [8,9]. There it developed major epidemics in the 1900s that are among the worst in agricultural history [10], linking its most prone geographical area to its colloquial name: Panama disease. It was only in 1910 that the soil-borne fungus *Fusarium oxysporum* f.sp. *cubense* (Foc) was identified as the causal agent in Cuba, from which the name of the *forma specialis* was derived [10].

## Genetic Diversity of *Fusarium oxysporum* f.sp. *cubense*, the Causal Agent of Panama Disease

**Fig 1:**
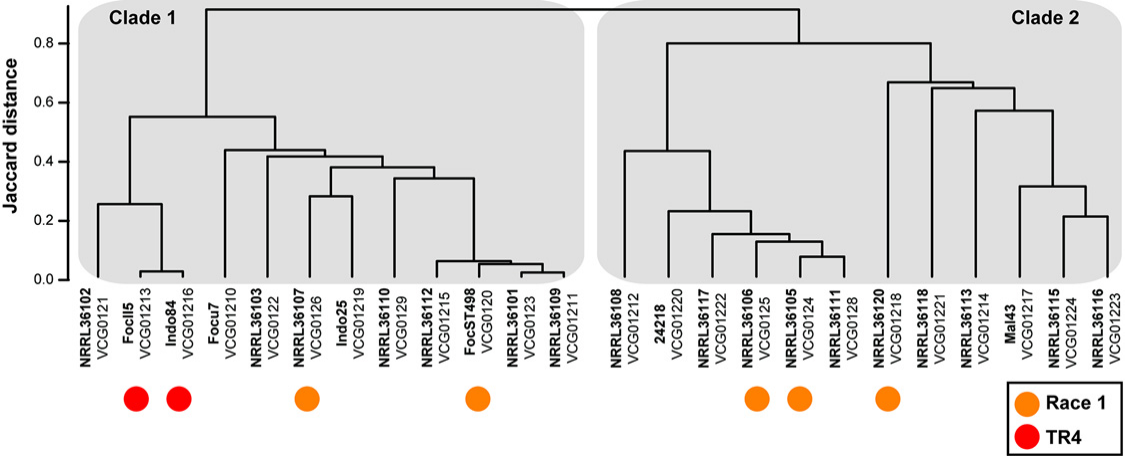
Genetic diversity of the banana pathogen *F*. *oxysporum* f. sp. *cubense*. Genotyping-by-sequencing analyses of the hitherto identified 24 vegetative compatibility groups (VCG) in *F*. *oxysporum* f. sp. *cubense* resulted in 12,978 DArTseq markers that divide Foc into two distinct clades—clade 1 and clade 2. VCG01216 is considered the same as VCG01213 [13]. The labels for race 1 isolates are based on personal communications with I. Buddenhagen and M. Dita. Although VCG01213 contains all TR4 isolates that cause the current Panama disease epidemic in Cavendish bananas, VCG0120—which has also been considered as race 4 [5]—and VCG0124 [36] have also been recovered from symptomatic Cavendish plants.

Foc belongs to the *F*. *oxysporum* species complex: a suite of asexual, morphologically similar, pathogenic and non-pathogenic strains affecting a wide variety of crops [11]. Foc likely co-evolved with its host species *Musa* in its center of origin [12–15]. Traditionally, phenotyping has identified three Foc races (1, 2, and 4) that cause disease in different subsets of banana and plantain cultivars [5,8]. However, Foc race designations are cumbersome and hence other methods unveiling genetic diversity were developed. Vegetative compatibility group (VCG) analyses largely divide Foc into 24 unique VCGs (VCG0120 through VCG0126 and VCG0128 through VCG01224) [5,13,16]. Later, DNA markers revealed the polyphyletic origin of Foc, as some VCGs are taxonomically closer to other *F*. *oxysporum* formae speciales than to other Foc VCGs [12,14,17]. Moreover, strains belonging to diverse VCGs infect particular banana cultivars and, hence, were grouped in the same race, suggesting that pathogenicity towards a specific cultivar evolved either convergently [5,12,14] or resulted from horizontal gene transfer among members of the *F*. *oxysporum* complex [18]. Overall, Foc lineages show a remarkable dichotomy, referred to as types or clades [12–14,19–22]. High-resolution genotyping-by-sequencing analyses using DArTseq—which generates short sequence reads after a genome-wide complexity reduction through restriction enzyme digestion [23]—validate and extend these findings (Fig 1). Based on genome-wide DArTseq markers, 24 Foc strains (representing all hitherto known VCGs) split into two groups. These largely corroborate the aforementioned clades, except for VCG0123 [13,14,20,22], VCG01210 [19], VCG01212 [20], and VCG01214 [21], which were occasionally reported in opposite clades, and VCGs 01221 to 01224, which were never classified before but now clearly belong to clade 2 (Fig 1).

Unfortunately, it is not well known which VCGs (the so-called Foc race 1 strains) caused the Panama disease epidemic in “Gros Michel” and, hence, their geographical dissemination is still unclear (I. Buddenhagen and M. Dita, personal communications). The current epidemic in Cavendish bananas, however, is caused by VCG01213 [5], colloquially called Tropical Race 4 (TR4).

## Panama Disease: History Repeats Itself

Large railway projects in Central America in the late 1800s facilitated industrial banana production and trade [10], which was entirely based on “Gros Michel” bananas [8]. The unparalleled vulnerability of “Gros Michel” to race 1 strains drove aggressive land-claiming policies in order to continue banana production. However, this did not stop the epidemic as Panama disease was easily entering these new areas through infected planting material. Hence, by the 1960s, the epidemic reached a tipping point with the total collapse of “Gros Michel” [9]. Fortunately, there was a remedy: Cavendish bananas—maintained as interesting specimens in botanical gardens in the United Kingdom and in the United Fruit Company collection in Honduras—were identified as resistant substitutes for “Gros Michel.” A new clone was “born” that, along with the new tissue culture techniques, helped save and globalize banana production [5,8,9].

However, in the late 1960s, Panama disease emerged in Cavendish bananas in Taiwan, but TR4 was only identified as its cause in 1994 [9,24,25]. Surprisingly, this initial outbreak did not awaken the banana industry and awareness levels remained low, despite the lack of any Cavendish replacement that met market demands and the susceptibility of many local banana cultivars to TR4 [5] (see also http://panamadisease.org/en/news/26). Thus, TR4 threatens not only the export trade but also regional food provision and local economies.

## Tropical Race 4, a Single Pathogen Clone, Threatens Global Banana Production

Ever since TR4 destroyed the Cavendish-based banana industry in Taiwan, its trail in Southeast Asia seems unstoppable with incursions and expansions in the Chinese provinces of Guangdong, Fujian, Guangxi, and Yunnan as well as on the island of Hainan. Since the 1990s, TR4 has also wiped out Cavendish plantations in Indonesia and Malaysia; between 1997 and 1999, it significantly reduced the banana industry near Darwin in the Northern Territory of Australia. It was first observed in the early 2000s in a newly planted Cavendish banana farm in Davao (on island of Mindanao, Philippines), where it currently threatens the entire banana export trade [26]. Since 2013, incursions outside Southeast Asia were reported in Jordan [27], Pakistan, and Lebanon [28], informally announced in Mozambique and Oman, and just recently noted in the Tully region of Northern Queensland, Australia. By now, TR4 may have affected up to approximately 100,000 hectares, and it is likely that it will disseminate further—either through infected plant material, contaminated soil, tools, or footwear, or due to flooding and inappropriate sanitation measures [5,29]. Clearly, the current expansion of the Panama disease epidemic is particularly destructive due to the massive monoculture of susceptible Cavendish bananas.

**Fig 2:**
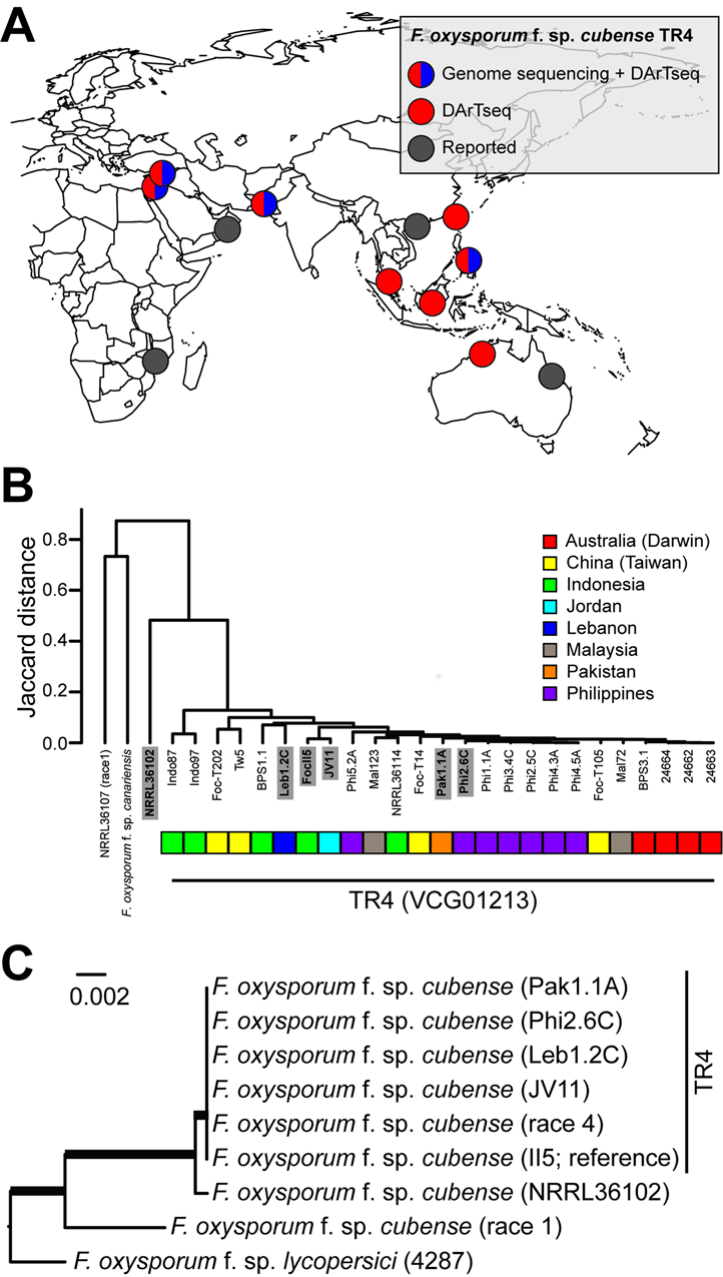
Phylogeography of *F*. *oxysporum* f. sp. *cubense* Tropical Race 4 (TR4). (A) Geographical locations of proclaimed TR4 incursions in Southeast Asia, Australia, Africa, the Middle East, and the Indian subcontinent. Different colors indicate if and how the genetic diversity of collected isolates was assessed. (B) Limited genetic diversity between multiple Foc TR4 isolates from distinct geographical locations revealed by hierarchical clustering, based on 4,298 DArTseq markers. Countries of origin for each of the TR4 isolates are indicated by different colors. (C) Phylogenetic analysis of selected Foc TR4 isolates (highlighted in bold in panel B) and related *F*. *oxysporum* species, based on whole-genome re-sequencing data. Phylogenetic tree analysis was performed using REALPHY [37], applying the PhyML algorithm for tree constructing (Foc II5 reference genome). The *F*. *oxysporum* f.sp. *lycopersici* and the *F*. *oxysporum* f. sp. *cubense* II5 genomes, as well as Foc race 4 and race 1 genomes, are publicly available at GenBank (http://www.ncbi.nlm.nih.gov/genome/genomes/707). Robustness of the grouping was assessed by 500 bootstrap replicates, and thick branches indicate maximum support.

Foc is a haploid asexual pathogen [8] and is therefore expected to have a predominantly clonal population structure [13,14,19–22]. Comparison of re-sequencing data of TR4 isolates from Jordan, Lebanon, Pakistan, and the Philippines—with the publicly available reference genome sequence of Foc TR4 strain II-5 (http://www.broadinstitute.org/)—indeed shows a very low level of single nucleotide polymorphisms (SNPs) (about 0.01%). This, together with a highly similar set of DArTseq markers, suggests that the temporal and spatial dispersal of TR4 is due to a single clone (Fig 2). This finding underscores the need for global awareness and quarantine campaigns in order to protect banana production from another pandemic that particularly affects vulnerable, small-holder farmers.

## Strategies for Sustainable Panama Disease Management

Any disease management eventually fails in a highly susceptible monoculture. Managing Panama disease with its soil-borne nature, long latency period, and persistence once established is, therefore, impossible without drastic strategy changes. Evidently, exclusion is the primary measure to protect banana production, which requires accurate diagnosis based not only on visual inspection, as this overlooks important aspects of its genetic diversity and epidemiology. New molecular-based diagnostics rapidly detect TR4 in (pre)symptomatic plants [30], soil, and water and, hence, can be used for surveillance and containment, which are key to avoiding an encounter of TR4 with Cavendish monocultures. Additionally, a thorough understanding of Foc epidemiology and pathology is urgently required, as this facilitates developing effective methods to destroy infected plants and (biological) soil treatments, thus reducing the inoculum quantity. Furthermore, we showed that high-throughput genome analyses unveil Foc population diversity (Figs 1 and 2), rather than lengthy and cumbersome VCG analyses, which enables resistance deployment strategies. Finally, effective disease management cannot be achieved without adequate disease resistance levels. “Cavendish”-based somaclones [31] do not satisfy local or international industry demands (apart from the epidemiological risks), as this germplasm is, at most, only partially resistant to TR4 [32]. Instead, the substantial genetic diversity for TR4 resistance in (wild) banana germplasm, such as accessions of *Musa acuminata* ssp. *malaccensis* [4], can be exploited in breeding programs and/or along with various transformation techniques [33–35] to develop a new generation of banana cultivars in conformity with consumer preferences. Developing new banana cultivars, however, requires major investments in research and development and the recognition of the banana as a global staple and cash crop (rather than an orphan crop) that supports the livelihoods of millions of small-holder farmers. Until new, commercially viable, and resistant banana cultivars reach markets, any potential disease management option needs to be scrutinized, thereby lengthening the commercial lifespan of contemporary banana accessions. The current TR4 epidemic and inherent global attention should be the wake-up call for these much needed strategy changes.

## Supporting Information

S1 Table Isolate collection at Wageningen University and Research Center used in this study. (XLSX)
